# Modeling of the Flow Field and Clad Geometry of a Molten Pool during Laser Cladding of CoCrCuFeNi High-Entropy Alloys

**DOI:** 10.3390/ma17030564

**Published:** 2024-01-25

**Authors:** Dachuan Tian, Chonggui Li, Zhiguo Hu, Xintong Li, Yajun Guo, Xiaosong Feng, Zhenhai Xu, Xiaoguang Sun, Wenge Li

**Affiliations:** 1 School of Materials Science and Engineering, Shanghai University of Engineering Science, Shanghai 201620, China; 2 Shanghai Aerospace Equipment Manufacturer Co., Ltd., Shanghai 200245, China; 3 National Key Laboratory for Precision Hot Processing of Metals, Harbin Institute of Technology, Harbin 150001, China; 4 Technical Engineering Department, CRRC Qingdao Sifang Co., Ltd., Qingdao 266111, China; 5 Institute for Marine Materials Science and Engineering, Shanghai Maritime University, Shanghai 201306, China

**Keywords:** laser cladding, molten pool flow, high-entropy alloys, clad geometry, simulation

## Abstract

A flow field analysis was performed in this research using the ANSYS Fluent module, and a dynamic heat source employing UDF was constructed using the DEFINE_PROFILE macro. A VOF model was developed to track the volume fraction of each fluid throughout the computational domain as well as the steady-state or transient condition of the liquid–gas interface in the free liquid surface area. To determine the distribution state and regularity of the molten pool flow field, the flow field velocity was calculated iteratively by linking the Simple algorithm with the horizontal set method. The molten pool was concave, indicating that the key hole was distributed narrowly. Inserting cross-sections at different depths yielded the vector distribution of the molten pool flow velocity along the depth direction. We set up monitoring sites along the molten pool’s depth direction and watched the flow change over time. We investigated the effects of the process parameters on the flow field’s vector distribution.

## 1. Introduction

304 stainless steel has great corrosion resistance, can be used in most acidic situations for a long time, and has a high strength and toughness [1,2,3]. It can also survive the erosion caused by various corrosive media at room temperature, such as oxidation, alkalinity, and high temperature, and is, thus, widely used in food processing equipment, pressure vessels, chemical equipment, medical equipment, building decorations, car parts, and in other sectors. However, the low hardness and poor wear resistance of 304 stainless steel severely limit its industrial applications [4]. As a result, the application of a high-entropy protective coating to the surface of stainless steel can significantly extend the service life of severe wear strips under high-temperature settings. There have been few studies on how to improve the performance of 304 stainless steel with high-entropy alloy coatings.

High-entropy alloys are significantly better than conventional metals because of their high hardness, high toughness, and high thermal stability as well as their good wear resistance, corrosion resistance, and other factors [5]. Under some special conditions, high-entropy alloys can even break the limits of existing materials, so they have become a hot spot within the development of material science [6,7,8]. Therefore, the preparation of a high-entropy protective coating on the surface of stainless steel could effectively extend the service life of severe wear strips under high-temperature conditions.

The process parameters typically used have a significant impact on the shape and coating quality of the final clad layer. The parameters of the laser cladding process include the laser power, scanning speed, defocus amount, protective gas, and so on. The laser power determines the temperature and depth of the covered area, which usually needs to be adjusted according to the material type and thickness. The scanning speed affects the speed of movement on the material’s surface and affects the thickness and compactness of the cladding layer. Goodarzi et al. [9] analyzed the influence of laser cladding process parameters on the geometry of the composite layer and analyzed the cladding results, concluding that the laser power and cladding speed are the main parameters in controlling the width of the cladding layer. Hofman et al. [10] used a novel method to determine the envelope geometry. The correlation between the observable melt pool characteristics and dilution was investigated using this model. Different combinations of the cladding speed, laser power (distribution), and substrate temperature were simulated. The simulation results were compared to the experimental results with high agreement. Benarji et al. [11] performed a finite element analysis of the L-MD to understand the thermal behavior governing the microstructural features (grain size and morphology). The input parameters of the L-MD were the effects of the scanning rate, powder feeding rate, and laser power on the cladding height and width and the variation in the solidification rate and temperature gradient under various process parameters. The composite width and height increased significantly with the laser power and the powder feeding rate but decreased with the scanning rate.

The laser melting process involves a high-temperature melting and rapid cooling process, which results in a harsh environment that is difficult to monitor. The laser cladding process is accompanied by a variety of heat exchanges, such as heat conduction, heat convection, and heat radiation, and the thermal change is difficult to analyze through experiments, so the use of a numerical model that reproduces the physical process can reduce the experimental period and cost [12]. Afshari et al. [13] used finite element techniques to simulate the laser cladding process to assess the effects of the scanning rate and laser power on the changes in the microstructure, geometry, and temperature of Inconel 718. It was shown that by increasing the scanning rate and decreasing the laser power, the height and length decreased, as the heat transfer to the sample was faster than its temperature rise speed. Hao et al. [14] constructed an adaptive cladding layer and mobile heat source models through an inverse modeling method, which realized the temperature distribution of laser cladding with different process parameter combinations, and they verified the effectiveness of the proposed model through the numerical and experimental results. Khomenko et al. [15] developed a new, coupled kinetic model of heat transfer and coagulation for optimizing microstructures in laser additive manufacturing applications. Their developed model had a fairly good agreement with the experimental data. In this paper, the influence of the processing parameters on the orbital macroscopic and microscopic parameters are analyzed. A method for changing the average crystal size and simultaneously preserving the orbital height and width is proposed.

## 2. Mathematical Modeling of Single-Track Laser Cladding

### 2.1. Model Building and Meshing

**Figure 1:**
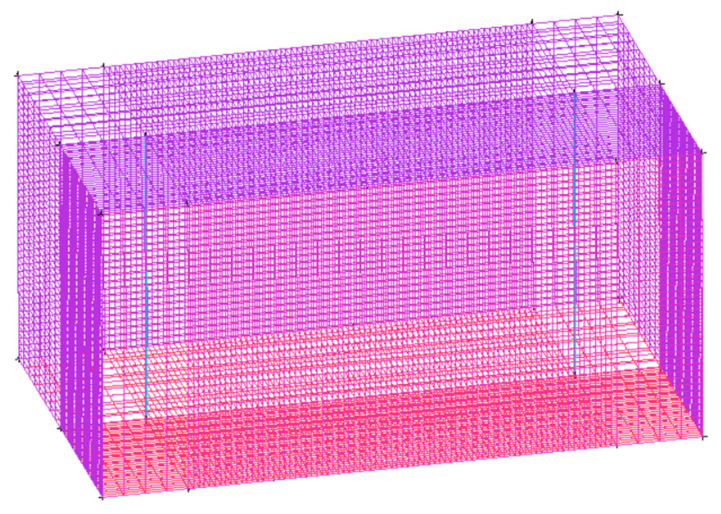
Model geometry and grid.

The cuboid model developed in this study was established using the ICEM CFD module in the ANSYS 18.2 software, as shown in Figure 1. The lower layer was a stainless-steel substrate, and the powder layer reflow field in the upper layer became an area of free liquid level change. Because the substrate powder is basically symmetrical in the actual cladding process, it could be divided from the symmetry surface, and half of the model could be built to reduce the calculation amount in the simulation process. After establishing the basic model, the six faces of the model needed to be named to impose the boundary conditions on each face using the Fluent 18.2 software. After naming the six sides of the model, the edges were linearly cut at the absolute value through the Blocking module. The closer the mesh is to the molten pool, the denser the mesh division is, so the edges of the near seam area needed to be divided into more nodes, and each segment was 0.1 mm. The partition grid could be previewed in Pre-mesh. The model had 100,560 units. If the preview grid met the requirements, the formal grid was generated through the Load from the Blocking module, and the model was exported. The process parameters of this simulation test are shown in Table 1. The total time step calculated by the simulation was 1 s, and the sub-time step was 0.001 s.

### 2.2. The User-Defined Function (UDF) Loads the Heat Source Model

The UDF is a user−defined function that can be passed to the solver, and is essentially a set of macros, each with its own role. The compilation of the UDF can complete many of Fluent’s dynamic processes, such as the loading of heat sources. The wall heat source of the laser cladding process is not static but moves along the direction of the laser scan. Firstly, the heat flux density of the laser heat source is not uniformly distributed (it can be Gaussian, biellipsoidal, annular heat source distribution, etc.); secondly, the heat source sweeps across the plane, changing the heated area in the space. In this scenario, the heating surface could not be simply defined as a constant heat flux density, and the UDF must specify the thermal wall surface to simulate the scanning heating of the heat source. This simulation applied a dynamic heat source by writing a UDF via the DEFINE_PROFILE macro.

When using the attenuation heat source model to solve the problem, a heat source outside the molten pool is present. The heat generation area in the molten pool is larger the closer it is to the thermal escape surface, while the heat generation area outside the molten pool is smaller. However, this is inconsistent with reality. Therefore, a Gaussian rotating heat source model with the heat flow changing with the depth was selected. The function expression is shown in the following equation [16]:

(1) $$q\left(x,y,z\right)=\frac{3\mathrm{MQ}}{\pi H\left(1-\frac{1}{e^{3}}\right)}\exp\left(-\frac{3M}{\log\left(\frac{H}{z}\right)}\left(x^{2}+y^{2}\right)\right)$$

where H is the heat source height, Q is the heat input rate, and M is the heat source concentration coefficient.

The heat source concentration coefficient M is a function of the heat source radius correlation, as follows:

(2) $$M=\frac{3}{R_{0}}$$

### 2.3. The Underlying Assumptions of the Model

Fluid movement is a complex physical process in practical problems. There are many factors affecting the viscosity and compressibility of a fluid. In the process of laser melting, the interaction process between the laser beam and the powder layer and substrate is also a complex process. Therefore, to simulate the model, certain assumptions must be made:

1. This article ignored the thermal recoil pressure of metal evaporation and the influence of the protective gas on the free interface of the melting tank.
2. The influences of the surface tension of the molten pool and the recoil pressure on the morphology and flow of the molten pool were considered.
3. The liquid in question was an incompressible Newtonian liquid, and the molten pool liquid exhibited laminar flow. The material was isotropic, and its heat did not vary with the position.

## 3. The Volume of Fluid (VOF) Model and the Simple Algorithm

### 3.1. VOF Model

To simplify the model, we only considered the melting tank and air phases. The numerical analysis of the interface between two insoluble fluids is typically handled using the Lagrangian and Euler methods [17]. Compared with the Lagrangian method, the Euler method addresses the significant interface deformation that occurs during laser melting. The Euler method encompasses several techniques, including the phase field method, the horizontal set method, and the diffuse reflection interface method. The selected method was the VOF method. The VOF model was used to divide the fluid into small cells (or grids) and define the volume fraction within each cell, representing the proportion of the volume containing a substance in that cell to the total volume. Then, the volume fraction within each cell was updated by calculating the mass and momentum transfer between each of the phases in the fluid. The velocity, pressure, and other physical parameters of the fluid were also calculated.

The VOF model was used to introduce the air-phase volume fraction α_g_ and the alloy-liquid-phase volume fraction α_A_. the following constraints also needed to be met for the two-phase volume fraction:

(3) $$\alpha_{g}+\alpha_{A}=1$$

The interface between the two phases can be tracked by solving the continuity equation for their volume fraction, as described in the following equation [18]:

(4) $$\frac{1}{\rho_{A}}[\frac{\partial }{\partial t}(\rho_{A}\alpha_{A})+\nabla \cdot ({\rho_{A}\alpha }_{A}\vec{v})]=S_{\alpha_{g}}+\sum_{\alpha_{A}=1}^{n}\left(\dot{m_{gA}}-\dot{m_{Ag}}\right)$$

where ρ_A_ is the alloy liquid density, $\vec{v}$ is the fluid flow rate, $\dot{m_{gA}}$ is the mass transfer from the gas phase to the alloy liquid phase, and $\dot{m_{Ag}}$ is the mass transfer from the alloy liquid phase to the gas phase.

According to the volume fraction of one phase, it is possible to determine the corresponding phase of cell. This can be achieved as follows:

(5) $$\alpha_{A}=1\;--\;Liquid\;phase$$

(6) $${0<\alpha }_{A}<1\;--\;Air\;and\;liquid\;border$$

(7) $$\alpha_{A}=0\;--\;Gaseous\;phase$$

**Figure 2:**
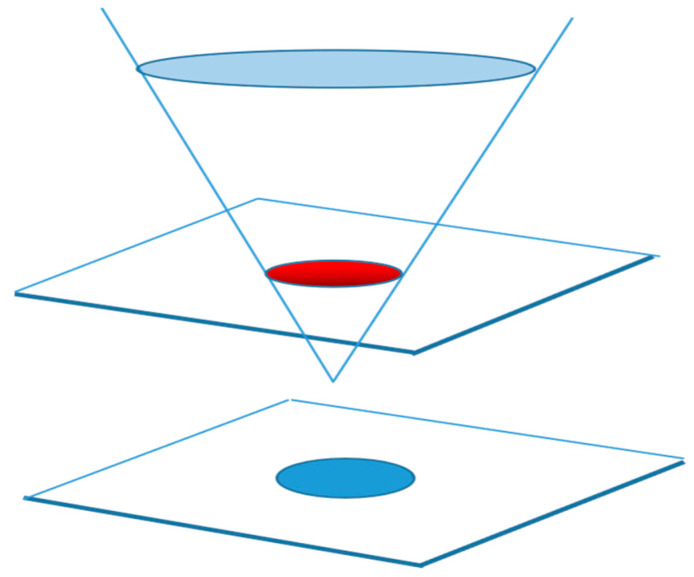
Schematic diagram of the level set.

However, it is difficult to solve the complex discontinuity fluid problem. In order to solve the problem of discontinuity interface crossing, we used the horizontal set method in conjunction with the VOF model. The VOF model was coupled with the horizontal set method in laser cladding to investigate the flow behavior of the melting tank. This was achieved by tracking the liquid–gas interface between the air and the melting tank. The horizontal set method (level set) is a technique for analyzing curve or surface evolution. It was first proposed by Osher and Sethian in 1988. Since then, it has become a popular technique in the fields of computational fluid mechanics, image processing, and geometric modeling. The scalar function (horizontal set function) is used to define the position and shape of the fluid interface. The fluid interface is represented by the isosurface of the horizontal set function, as shown in Figure 2. The interface of the two phases is indicated by the red ellipse. The simulation of the motion and deformation of the fluid interface can be simulated by evolving the level set function. The evolution of the level set function is described by a partial differential equation, or the level set equation, a partial differential equation that uses curvature flow to drive its evolution. The equation for the horizontal set can apply force on a fluid interface or curve, causing it to move along the gradient direction and adjust its shape based on the curvature.

### 3.2. Simple Algorithm

Solving the three major control equations in fluid mechanics, namely the mass equation, the momentum equation, and the energy equation (the generalized N-S equation), is a challenging task. The difficulty in solving the N-S equation mainly arises from the following three points: (1) the N-S equation contains three velocity field equations, namely Ux, Uy, and Uz, but it lacks the corresponding pressure equation. (2) The momentum equation is also obtained through the continuous equation constraint as the three velocity field equations are solved. (3) In the case of incompressible isothermal flow, the density and temperature remain constant and cannot be determined through the equation of state. To address the computational challenges with the N-S algorithm, the Simple algorithm was used for this simulation. The Simple algorithm [19] is an iterative method used to solve the pressure and velocity components of the N-S equation. The algorithm’s core involves two steps: (1) deriving the pressure equation from the momentum equation and the continuity equations and (2) correcting the velocity field to satisfy the continuity equation. The process is as follows:

1. The velocity field is first solved by the momentum equation, where the velocity does not satisfy the following continuous equation:
  (8) MU=−∇p
2. The pressure field is solved by Poisson’s ratio with the following formula:
  (9) $$\nabla \cdot (A^{-1}\nabla p)=\nabla \cdot (A^{-1}H)$$
3. After obtaining the pressure field, the velocity field can satisfy the continuous equation.

## 4. The Force of the Molten Pool

### 4.1. Recoil Pressure of the Molten Pool

During the process, the metal liquid will vaporize when it reaches boiling point. This will create a reverse pressure on the molten pool, causing its surface to sink. The laser can then directly hit the bottom of the pit to create a thin and narrow shape of the molten pool. This process continues until the recoil pressure reaches a dynamic balance with the surface tension and gravity of the liquid metal. The pressure resulting from the recoil acts on the wall surface of the hole perpendicular to the air–liquid interface. Semak V. proposed the recoil pressure model [20] as follows:

(10) $$P_{r}=0.54P_{0}\exp\left(\Delta H_{v}\frac{T-T_{b}}{\mathrm{RT}T_{b}}\right)$$

In the formula, $P_{0}$ is the ambient pressure, $\Delta H_{v}$ is the evaporative and latent heat of the material, T is the Spoon hole wall temperature, $T_{b}$ is the boiling point of the material, and R is the ideal gas constant.

### 4.2. Heat Buoyancy of the Molten Pool

The buoyancy of the pool thermal energy is typically combined with natural or forced convection and is influenced by several factors, including the welding parameters, material properties, environmental conditions, etc. During the melting process, the flow rate of the molten pool is slow, and the flow area is small; thus, the Boussinesq assumption is used. The Boussinesq assumption is based on the buoyancy effect of a small change in fluid density, which creates a buoyancy perpendicular to the density gradient. This buoyancy can be calculated by introducing a term called the Boussinesq approximation:

(11) $$F=-\rho_{0}\beta \left(T-T_{0}\right)g$$

In the formula, $\rho_{0}$ is the density of fluid, β is the volume expansion system, and $T_{0}$ is the reference temperature.

### 4.3. The Surface Tension of the Molten Pool

During laser melting, the flow behavior of the molten pool is largely dependent on the magnitude and direction of the surface tension. The surface tension flow is caused by a surface tension gradient. As the temperature increases, the surface tension constantly decreases, and the two are negatively correlated. When the temperature gradient is generated, the surface tension gradient of the molten pool also follows. Therefore, the calculation of the surface tension can be simplified using the following equation:

(12) $$\sigma =\sigma_{m}^{0}+\frac{d\sigma }{\mathrm{dT}}\left(T-T_{m}\right)$$

$where\sigma_{m}^{0}$ is the surface tension of the pure metal at the melting point, and $T_{m}$ is the melting point of the metal.

## 5. Results and Discussion

### 5.1. Dynamic Evolution of the Flow Field of Laser-Coated High-Entropy Alloys

**Figure 3:**
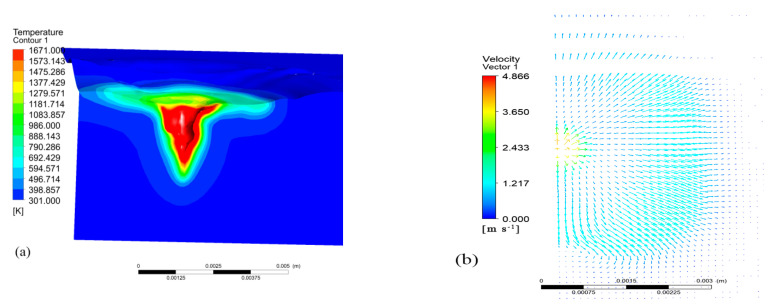
(**a**) Morphology of the molten pool; (**b**) vector distribution of flow velocity on the surface of the molten pool.

The morphology of the power of 2500 W and the surface flow velocity vector distribution with a scanning speed of 10 mm/s are shown in Figure 3a. The temperature range was 301–1671 K, which corresponded to the room temperature and the melting temperature of the metal substrate. The molten pool was concave, resembling a spoon with a wide top and narrow bottom. The material was rapidly vaporized by the laser, producing a pressure that emitted the molten metal and formed a spoon-shaped hole. The laser melted and evaporated the surface of the material. If the evaporation speed is large enough, the steam recoil pressure can overcome the tension of the liquid metal surface and liquid gravity, causing the molten pool in the liquid metal at the laser zone to become concave and form a small pit. The beam acted directly at the bottom of the pit, causing the metal to melt and gasify further. The high-pressure vapor then forced the liquid metal around the molten pool, deepening the hole. This process continued until a hole was formed in the liquid metal. The temperature at the center of the molten pool was the highest, and it spread to all sides. When the metal vapor pressure generated by the laser beam in the hole reached equilibrium with the surface tension and gravity of the liquid metal, a stable hole was formed without further deepening. The maximum flow rate was observed at the center of the molten pool due to the combined action of the recoil pressure and surface tension, gradually decreasing towards the periphery. When the flow rate of the molten pool decreased to a certain extent, the liquid metal moved from the center of the laser beam to the periphery of the molten pool due to the negative temperature coefficient of the surface tension. As a result, the flow rate of the molten pool rose again, exhibiting a wave-like pattern.

### 5.2. Distribution of Molten Pool Flow Fields at Different Depths

**Figure 4:**
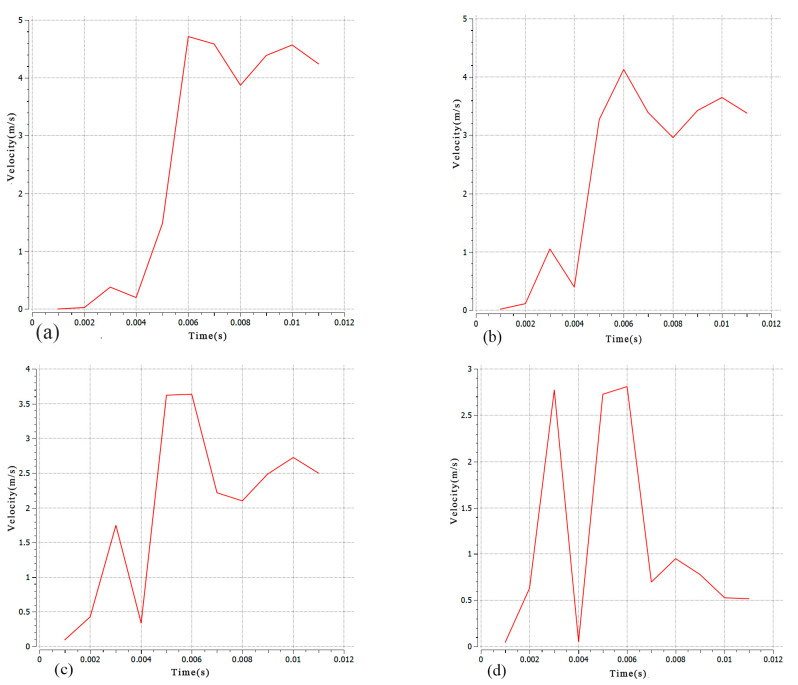
Time process curves of vector at the molten pool center in the depth direction (**a**–**d**).

In order to study the flow field distribution within the steady-state melting tank, this simulation observed the symmetrical cross-section of the molten pool. Monitoring points A, B, C, and D were selected at different depths of the molten pool, and the velocity change curve over time is shown in Figure 4. It is evident that the molten pool had a flow rate of 0 before t = 0.001 s, indicating its initial formation at this time. The flow rate of the molten pool increased over time and reached its peak at t = 0.006 s. Subsequently, the monitoring point gradually decreased, with a faster decline rate closer to the bottom of the molten pool. The laser’s rapid heating caused a decrease in the molten pool temperature gradient and the liquid surface tension, resulting in a sudden decrease in the flow rate of the detection point at t = 0.003 s. The decrease was closer to the bottom of the molten pool. The maximum flow rate decreased as the molten pool depth increased. This was due to the greater friction force between the unmelted substrate and the molten pool fluid, which hindered the liquid flow as the pool approached the bottom. The flow rate in the upper part of the molten pool exceeded both the recoil pressure and the surface tension.

**Figure 5:**
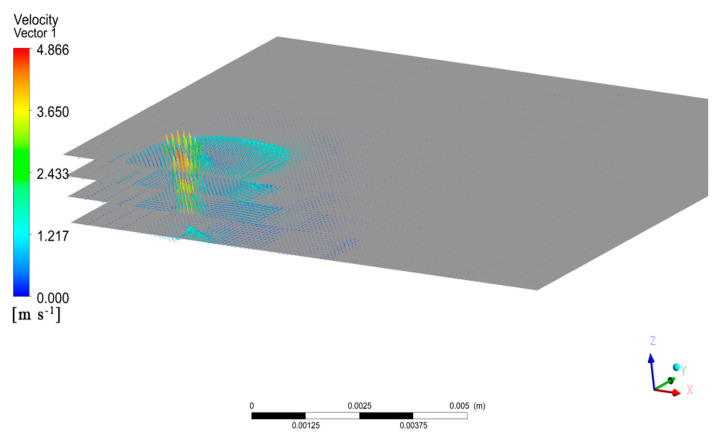
Distribution of molten pool flow fields with different depth cross-sections.

To investigate the distribution of the flow field inside the molten pool, we obtained the velocity vector distribution of the molten pool flow along the depth direction, as shown in Figure 5. As the section depth decreased, the overall velocity of the molten pool slowed significantly, and the wavy velocity distribution caused by the surface tension gradually disappeared. The surface tension of the molten pool section increased as it approached the heat source due to its proximity to the heat source and the higher temperature. Each section of the molten pool had an upward flow trend, which weakened as the section depth decreased continuously.

**Figure 6:**
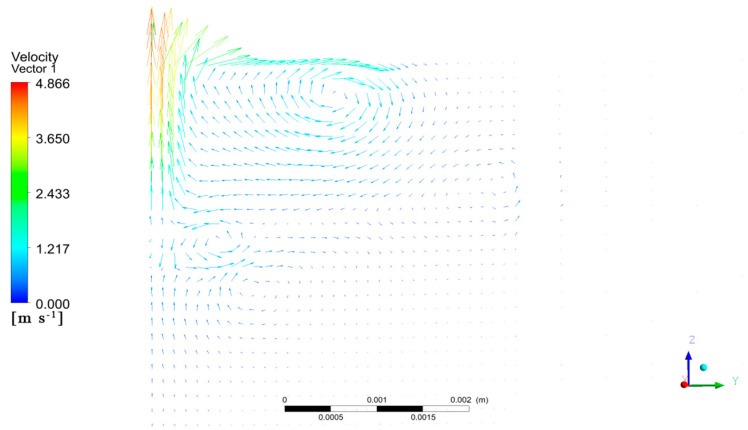
Vector maps of fluid velocity on longitudinal plane of molten pool.

A plane perpendicular to the laser scanning direction was inserted at the center of the molten pool, and its flow rate distribution vector is shown in Figure 6. On the far-left-hand side of the vector distribution map, there is a symmetric line on the symmetry surface. This line reflects the flow rate distribution at the center of the molten pool. The molten pool had vortices of varying sizes distributed along the symmetry line. The flow rate of the molten pool corresponded to the distribution of the molten pool flow field in different cross-sections. This distribution was affected by the large surface tension and recoil pressure, causing the vortex at the bottom and closer to the center of the pool to have a greater flow rate. Technical terms are explained when first used.

### 5.3. Effect of Laser Power on the Flow Field of Laser-Coated High-Entropy Alloys

**Figure 7:**
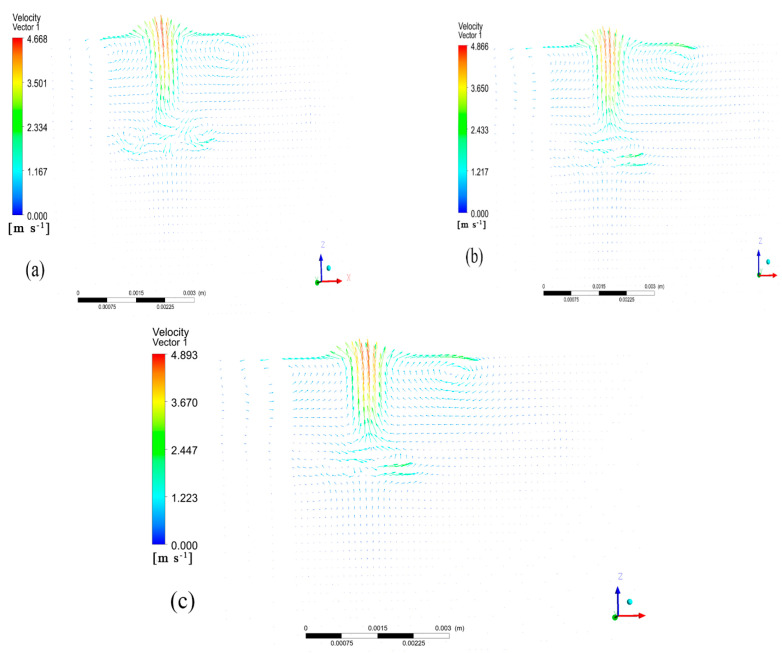
Vector maps of fluid velocity on the symmetry plane of molten pool at different laser powers: (**a**) *p* = 2000 W, (**b**) *p* = 2500 W, (**c**) *p* = 3000 W.

The convection within the molten pool occurred primarily in the cross-section parallel to the laser scanning direction, specifically at the symmetric surface. In order to study the influence of the laser power on the molten pool flow field, three molten pool flow fields with laser powers of 2000 W, 2500 W, and 3000 W, all with a scanning speed of 10 mm/s, were selected for comparison, as shown in Figure 7.

The molten pool exhibited the highest flow rate at its top and the lowest at its bottom. At the bottom and top of the molten pool, two symmetrical vortices appeared on the left and right, respectively. The center of the molten pool is where the vortices intersected. The left vortex was primarily sustained by the residual heat from the scanning and the heat conduction from the front end. As a result, the flow vector distribution was diluted, and the maximum flow rate was lower than that of the right vortex. As the laser cladding power increased, the highest flow velocity in the molten pool also increased gradually. This was due to the fact that the laser cladding process was mainly affected by the recoil pressure and the liquid surface tension. As the laser power increased, the temperature of the hole surface also increased. This, in turn, increased the recoil pressure and liquid surface tension, enhanced the convection effect, and increased the flow velocity in the molten pool. At 2000 W, the vortex flow at the bottom of the molten pool was more pronounced. As the power increased, the temperature gradient difference at the bottom of the molten pool gradually decreased, weakening the convection at the bottom and resulting in a more stable flow field.

### 5.4. Effect of Scanning Speed on the Flow Field of Laser-Coated High-Entropy Alloys

**Figure 8:**
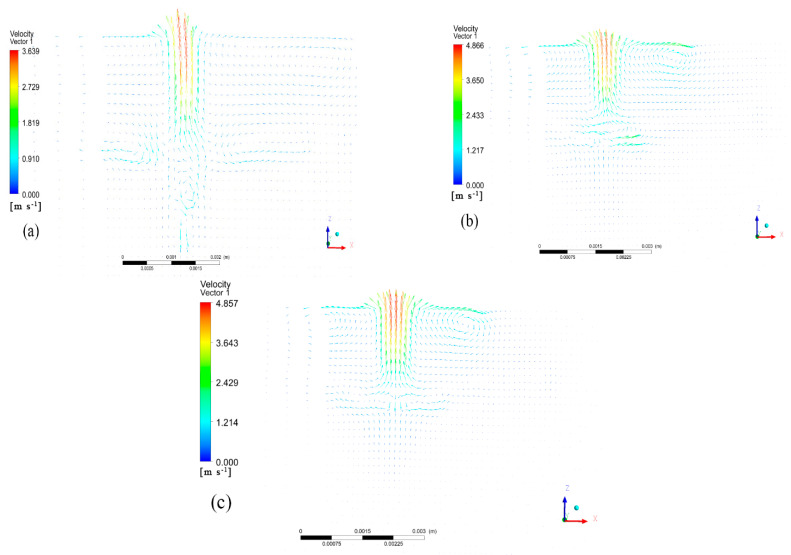
Vector maps of fluid velocity on the symmetry plane of molten pool under different scanning speeds: (**a**) v = 0.005 m/s, (**b**) v = 0.01 m/s, (**c**) v = 0.015 m/s.

The speed at which the scanning is conducted has an impact on the flow field of the high-entropy alloy melting cell during laser melting. An experiment was conducted using a laser power of 2500 W at three different speeds: 5 mm/s, 10 mm/s, and 15 mm/s. Figure 8 shows the distribution of the flow field and the velocity vectors at the interface of the molten pool. As the scanning speed increased, the heat input decreased, resulting in a smaller temperature gradient in the molten pool. Therefore, the size of the molten pool constantly decreased. Simultaneously, the increase in the scanning speed reduced the heat accumulation at the back end, causing the left vortex to continuously decrease. The maximum flow rates of the molten pool, as calculated from CFD-POST, were 3.5 m/s, 4.6 m/s, and 4.8 m/s, respectively. As the velocity of the laser sweep surface increased, the maximum flow velocity in the molten pool also increased. This was due to the heat accumulation decreasing, resulting in a reduction in the heat absorption per unit time. As a result, the temperature gradient increased, which in turn increased the inner surface tension of the molten pool, driving more intense convection at the top of the molten pool. The velocity vector at the bottom of the molten pool decreased as the scanning speed increased. At a speed of 5 mm/s, a vortex was clearly visible at the bottom of the molten pool. At a scanning speed of 15 mm/s, the convection effect at the bottom of the molten pool was negligible, and the fluid flow rate stabilized. A scanning speed that is too fast reduces the heat input, resulting in a smaller temperature gradient at the bottom of the molten pool and a decrease in the surface tension, which in turn leads to a smaller bottom flow rate.

### 5.5. Comparison with the Other Numerical Simulations

**Figure 9:**
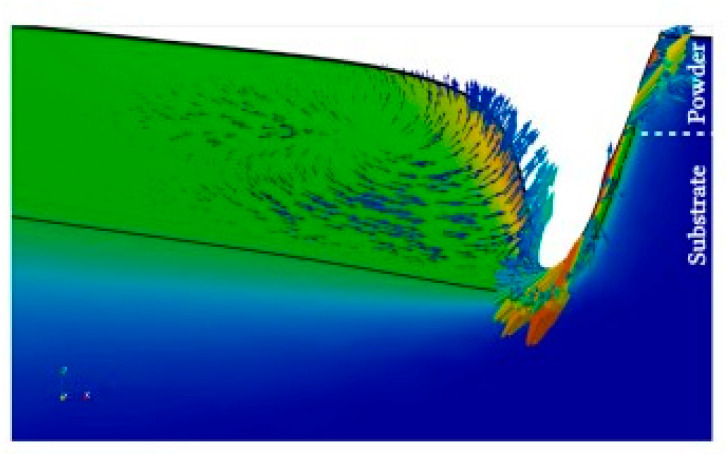
Flow velocity distribution at the pool interface [21].

Grange et al. [21] established a three-dimensional finite element model of Inconel 738 laser coating that considered the temperature, stress, and flow fields. The molten pool flow vector map at the interface was plotted, as shown in Figure 9. The molten pool had a wide keyhole shape at the top and narrowed at the bottom, and the maximum flow rate of the molten pool was at the top, and the flow rate decreased as the depth decreased. Compared to the simulation results presented here, the distribution of the fluid vectors in the molten pool was in agreement.

## 6. Conclusions

This paper presented an analysis of the flow law of fluid inside the molten pool under different processing parameters of the laser cladding flow field. It also investigated the influence law of the laser power and scanning speed on the flow field. The main conclusions are as follows:

1. At t = 0.001 s, the pool was formed. At t = 0.003 s, the flow rate suddenly decreased and then increased and reached a peak at t = 0.006 s. The molten pool exhibited a keyhole effect due to the recoil pressure, resulting in a wide and narrow hole. The surface flow velocity vector of the molten pool decreased initially and then increased in waves. Along the depth direction, the flow velocity at the top of the pool was much higher than at the bottom of the pool.
2. With decreasing the depth of the molten pool in different cross-sections, the overall flow rate of the molten pool slowed down. The wavy distribution shape gradually disappeared, and the molten pool had an upward flow trend. The trend became more intense closer to the top of the molten pool.
3. The velocity of the flow in the molten cell increased with the increase in the laser power, and the maximum flow velocity appeared at the top of the molten pool. When the laser power was low, there was a noticeable eddy current at the bottom of the melting pool. As the eddy current gradually dissipated, the flow rate at the bottom of the melting pool gradually stabilized.
4. With an increase in the scanning speed, the size of the melting pool decreased. Additionally, the left and bottom vortices of the melting pool also decreased, while the bottom flow velocity of the melting pool gradually decreased and the apical flow velocity increased.

## Figures and Tables

**Table 1:** Laser cladding process parameters.

Number	Laser Power (W)	Scanning Speed (mm/s)	Defocusing Amount (mm)	Spot Radius (mm)
1	2000	10	40	1
2	2500	10	40	1
3	3000	10	40	1
4	2500	5	40	1
5	2500	15	40	1

## Data Availability

The data presented in this study are available on request from the corresponding author.
